# Henry H. Cheek and transformism: new light on Charles Darwin's Edinburgh background

**DOI:** 10.1098/rsnr.2014.0038

**Published:** 2014-12-10

**Authors:** Bill Jenkins

**Affiliations:** Science Studies Unit, University of Edinburgh, Chisholm House, High School Yards, Edinburgh EH1 1LZ, UK

**Keywords:** Charles Darwin, Henry H. Cheek, transformism, evolution, University of Edinburgh

## Abstract

Evidence for the transformist ideas espoused by Henry H. Cheek (1807–33), a contemporary of Charles Darwin's at the University of Edinburgh, sheds new light on the intellectual environment of Edinburgh in the late 1820s and early 1830s. Cheek was the author of several papers dealing with the transmutation of species influenced by the theories of Étienne Geoffroy Saint-Hilaire (1772–1844), Jean-Baptiste Lamarck (1744–1829) and the Comte de Buffon (1707–88). Some of these were read to student societies, others appeared in the *Edinburgh Journal of Natural and Geographical Science*, which Cheek edited between 1829 and 1831. His writings give us a valuable window onto some of the transformist theories that were circulating among Darwin's fellow medical students in the late 1820s, to which Darwin would have been exposed during his time in Edinburgh, and for which little other concrete evidence survives.

## Introduction

As the pages of most scholarly biographies of Charles Darwin will attest, the Plinian Natural History Society of Edinburgh is often considered to have been a forum in which radical ideas about the natural world and humanity's place in it could be debated by its largely student membership. Adrian Desmond and James Moore have stated that the society's ‘meetings could be electric, while some topics bordered on the indictable.’^1^ Desmond and Moore recount the story of John Coldstream, an Edinburgh medical student and member of the society, as evidence of just how shocking the views expressed in the society's meetings could be. According to Desmond and Moore, the ‘Plinian debates on mind and matter threw him into a crisis’, leading to a complete mental breakdown during a visit to Paris after his graduation.^2^ Janet Browne is more measured in her assessment of the Plinian Society in her biography of Darwin. Although acknowledging that a ‘handful of members presented more radical thoughts to their friends’, for the most part the papers read to the society ‘were entirely conventional in focus: they dealt with the circulation of sea currents, the identity of new plants around Edinburgh, or the anatomy of marine animals found in the Firth of Forth.’^3^

It is beyond the scope of this paper to deal with the issue of how genuinely subversive the society's deliberations were in the context of the Edinburgh of the 1820s. However, as we shall see when I turn to look at the evidence of the society's minute books, it does seem to have been a forum in which relatively daring and speculative ideas could be aired and debated among a like-minded group of students. The idea that specifically concerns me here is the transmutation of species.^4^ It is well known that several figures in medical and university circles in Edinburgh in the 1820s were convinced transformists, or at least sympathetic to transformist ideas, including Robert Grant (1793–1874), Robert Knox (1791–1862) and perhaps even Robert Jameson (1774–1854), the professor of natural history at the university.^5^ The *Edinburgh New Philosophical Journal*, edited by Jameson, published a series of transformist articles in the late 1820s and early 1830s, some by Grant, others anonymous. One has been plausibly credited to Jameson by James Secord.^6^ However, aside from Grant, who was a member of the society, little has been known about the specific beliefs of the members of the Plinian Society on this important subject. Evidence for the advocacy of transformist theories in this place and time could throw light on the reception and dissemination of transformism in Britain more generally at this crucial period of the nineteenth century, as well as on the influences to which Darwin would have been exposed during his time at Edinburgh in 1825–27. Although this paper is a case study of one individual, its conclusions do, I believe, have considerably wider resonances.

Unfortunately there is little surviving evidence of the views and opinions of most of the individual members of the Plinian Society. Frustratingly little is known about the views expressed in its meetings beyond the, for the most part, extremely terse entries in the society's minute books. A significant number of the students went on to distinguished careers; among other notable members were William A. F. Browne (1805–85), who became superintendent of the Royal Lunatic Asylum of Montrose and a leading reformer in the treatment of the mentally ill; William Francis Ainsworth (1807–96), who became a noted geographer, geologist and explorer; and, of course, Charles Darwin himself. But with the exception of Darwin, very little evidence survives as to whether any of them went on to develop the radical ideas found in the minutes of the society outside its meetings. However, one important, but hitherto largely unknown, exception to this is a medical student from Manchester called Henry Hulme Cheek (1807–33).

Aside from several contributions to the minutes of the Plinian Society, which I will examine below, two other pieces of evidence survive that give a fascinating insight into Cheek's beliefs regarding the transmutation of species. The first is an essay read before the Royal Medical Society on 29 January 1830 entitled ‘On the varieties of the human race’. The second additional source of evidence is in the form of a short-lived scientific journal, the *Edinburgh Journal of Natural and Geographical Science*, edited by Cheek and William Francis Ainsworth and published between 1829 and 1831. This contains several articles that shed considerable light on Cheek's views and opinions. It is difficult to know how typical Cheek was among his fellow medical students in the late 1820s and early 1830s. Nevertheless, the evidence that has survived provides an engaging case study of the ideas and opinions of one medical student on the transmutation of species at a pivotal moment in the history of evolutionary thought. It may even shed some new light on the early development of Darwin's ideas: the two men would certainly have known each other; not only did their student careers at Edinburgh overlap, but they were both members of the Plinian Society and the Royal Medical Society of Edinburgh. Indeed, Cheek and Darwin were both elected to the Plinian Society on the very same day. According to the minutes of the society, between 5 December 1826 and 27 March 1827 they were both present at 15 of its meetings. Attendance could be as low as 11 members, and was never higher than 23 while Darwin was a member, so there is little chance that they did not know each other. However, before conducting a review of Cheek's contributions to the Plinian Society, I will present an overview of his Edinburgh career.

## Henry H. Cheek in Edinburgh

Henry H. Cheek was born in 1807 and was baptized at the Church of Saint Stephen in Salford on 1 October of that year.^7^ According to Derbyshire County records, his father, William Henry Cheek, was a solicitor of Tideswell, Derbyshire.^8^ From the Edinburgh University matriculation records we know that he studied in Edinburgh between 1826 and 1832, when he graduated MD. His dissertation was on theories of the function of the spleen (*De Variis Conjecturis quod ad Lienis Utilitatem*). As we have seen, he joined both the Plinian Natural History Society and the Royal Medical Society of Edinburgh. He was one of the four annual presidents of the Royal Medical Society in 1829–30, as well as secretary of the Plinian Society in the 1826–27 session. In 1829, along with his fellow medical student Ainsworth, he took the unusual step of starting his own journal, the *Edinburgh Journal of Natural and Geographical Science*, which, as noted above, ceased publication in 1831 after three volumes.

Cheek used his journal to launch a campaign against the Wernerian Natural History Society and its president and founder, Robert Jameson, the professor of natural history at Edinburgh and keeper of the university museum. The Wernerian Society, which had been founded in 1808, counted among its members most important figures in natural history in Scotland in the first half of the nineteenth century. However, according to Cheek, who was not himself a member, all was not well at the society. In a pamphlet responding to ‘Address to the Members of the Wernerian Natural History Society’, Cheek summed up his criticisms of the society and of Jameson:

I can declare that, during the four years of my residence in Edinburgh, I have been grieved to see the Museum of the University closed to the student who did not purchase certain nominal privileges at an exorbitant price, and, what was more disgraceful, the total uselessness of that establishment to the man of science;—I have felt indignant at the perusal of the syllabus of lectures which the Professor of Natural History puts into the hands of his pupils, and which is only calculated to delude; and I have beheld with disgust a coterie brooding like a night-mare over the Wernerian Natural History Society, till there was little remaining of it but the mockery cast by its name, upon opinions which are now only to be found in the pages of the history of error.^9^

In reply to Cheek's accusations, Patrick Neil, the Edinburgh printer and secretary of the Wernerian Society, accused Cheek of ‘doing all in his power (fortunately little) to hold up to contempt and infamy either its President or Secretary, or both, by the grossest imputations’.^10^ He also accused Robert Knox, who had supported Cheek, of gross ingratitude to Jameson, who had helped him in his early career. He mocked Cheek's pretentions, claiming that he ‘wishes to hold himself forth as the patron of naturalists here,—the Baron Cuvier of Edinburgh; and he seems desirous that the Saturday evening conversaziones of Gardner's Crescent should vie with the soirées of the Jardin des Plantes.’^11^

The records of this dispute gives us an invaluable window into Cheek's position in Edinburgh's natural history circles in the first years of the 1830s. It seems that Cheek was at the centre of a circle of younger natural historians who met for regular conversaziones at his house in Gardner's Crescent, from which he also published his journal. From this position he seems to have felt confident enough, perhaps unwisely, to challenge the authority of the Wernerian Society and of its president, Robert Jameson. It might be speculated that this controversy, in which Neill seems to have had the last word, may have led, directly or indirectly, to the winding up of Cheek's journal after its next volume and hastened his departure from Edinburgh after his graduation in 1832, but it is impossible to know for certain.^12^ What is certain is that the year after his graduation, having returned to his home town of Salford, Cheek died at the tragically young age of 26 years. He was buried at Saint Stephen's Church in Salford on 30 April 1833.^13^

## Cheek at the Plinian Natural History Society

The Plinian Natural History Society was founded in 1823 by a group of undergraduate medical students. According to a biography of John Baird, one of the founders, he and his associates, ‘feeling the want of a society where younger students of nature could meet and discuss their views freely among themselves, unawed by the older and more mature naturalists of the day, resolved to institute a society for the advancement of the study of natural history, antiquities, and the physical sciences in general.’^14^ In 1829 the *Magazine of Natural History* included the following description of the activities of the society:

The means which have been adopted for the prosecution of the views of the Society are, the reading of papers, debates, the formation of a museum and library, and excursions to the country, for the examination and collection of objects of natural history. Papers have been read on subjects connected with all the departments of natural science, more especially on the zoology, botany, geology, mineralogy, meteorology, and antiquities of Scotland.^15^

Although Jameson was an honorary member, he never attended any of the meetings of the society.^16^ When asked about the Plinian and his relationship with it by the commissioners of the Royal Commission on the Scottish Universities in 1826, he described it as a forum ‘where young men can discuss subjects which they have heard in the class of Natural History, or in the other classes where such subjects are considered.’^17^ While he stated that the society had been founded ‘under his countenance’, he denied that he had ‘any particular controul’ over it. However, he went on to affirm that, in his opinion, the members ‘distinctly confine themselves to the proper subjects of investigation’.^18^

The minutes of the society for June 1826 noted that a deputation to Professor Jameson could report on ‘the very flourishing condition of the society—that there are at present about 150 members on its list and that although we have no compulsory laws and impose no fines, we have had a good attendance of members all session and an abundant supply of papers.’^19^ The society continued to flourish and attract new members throughout the later 1820s, its weekly meetings between November and July regularly attracting about 20 members. As the outline of the society's activities above indicates, papers given at its meetings addressed a very wide range of topics, from mineralogy and ornithology to more controversial topics such as phrenology and the existence of a vital principle; papers were even read to the society on subjects such as apparitions and astrology. The eclectic interests of the members meant that papers given at the society could occasionally prove controversial. On 27 March 1827 William A. F. Browne presented a paper entitled ‘On organization as connected with life & mind’. In a famous and widely discussed incident the account of this paper was later struck from the minutes of the society by an unknown hand.^20^ The announcement that the paper was to be given was also deleted from the minutes from the previous week's meeting. If the intention of the unknown censor was to erase all trace of the paper from the minutes, he did his work badly, for the account of the paper is still clearly legible. Unfortunately, in the absence of further evidence, no firm conclusion can be drawn regarding the circumstances of the deletion. In any case, it seems that the materialist and anti-vitalist views expressed in the paper were controversial to some even within the society, although clearly such views could be and were openly debated. Browne's paper was accorded an unusually full account in the minutes. Most of the others are represented only by frustratingly vague and brief descriptions, such as ‘On the purposes of nature as Exemplified in some peculiar organization of the Animal Kingdom’ by William Rathbone Greg, read on 23 December 1828, which seems to have led to a lively debate and might well have contained equally inflammatory material.^21^

In 1829 the *Magazine of Natural History* could still report, ‘It will be unnecessary to remark on the flourishing state of this Society, when it is stated that it is at present composed of upward of 180 members.’^22^ However, the society went into a marked decline after 1830. The minutes for 6 March 1832 refer to the ‘declining state’ of the society,^23^ and in those for the next meeting on 3 April, Cheek, at that time the convenor of the Committee, reported that the Committee considered that ‘the Plinian Society is not in a condition to be continued any longer as a separate institution’, a situation that he ascribed to the ‘apparent apathy in this University to the cultivation of those Branches of natural science for the prosecution of which this Society was originally founded.’^24^ The society did, however, continue to meet at increasingly irregular intervals until 19 May 1835, after which there was a lapse of almost six years until January 1841, when the minutes note that since ‘there has been no meeting of the Society since 1835 and there was no prospect of any revival of it taking place, it was moved by Dr Spittal that the Society be dissolved.’^25^

Cheek was elected a member of the society on 28 November 1826, the same day as Darwin.^26^ He became an enthusiastic member, seldom missing a meeting throughout the late 1820s. On 2 January 1827 he was elected secretary, a post he held until April of that year. He gave his first paper, on ‘Intermittent & Reciprocating Springs’, on 6 February 1827.^27^ Continuing with the hydrological theme, on 13 March of the same year he read a paper on ‘Observations on the milky appearance of some spots of water in the Red Sea’.^28^ This was not an original piece but was an article by a certain Captain Charles Newland that had appeared in *Philosophical Transactions of the Royal Society* as long ago as 1772.^29^ On 17 April he read a mysterious communication ‘in which he described some observations made by Mr Ainsworth and himself, whereupon Mr Ainsworth had deduced certain inferences, into which it would be unnecessary to enter on this occasion as he promised to state them more fully hereafter.’^30^ Sadly it has not been possible to determine what exactly this strange communication referred to. On 20 November 1827 he read a paper on ‘The Literary History of Scrofula, bearing relation to its popular name of Kings Evil.’ Cheek concluded that ‘authorities support the opinion of the occurrence of cures from the Royal touch, the proximate cause of which might be attributed to the action of the imagination assisted by the principle of imitation.’^31^ His next paper, read on 11 March 1828, was a foray into invertebrate zoology, ‘On the Annelides Sedentaires [sedentary annelid worms] of La Marck’.^32^ Too much should perhaps not be read into Cheek's familiarity with the work of Jean-Baptiste Lamarck (1744–1829) here, because Lamarck was the foremost authority on invertebrate zoology in the 1820s, whose work in this area was widely respected and quoted by natural historians who had no truck with his transformist theories. Cheek now demonstrated an interest in antiquities. On 8 April 1828 he presented a collection of about 60 Roman coins to the society,^33^ and on 15 April he read a communication on ‘Encaustic Pavements a specimen of which from a church in Derbyshire he presented.’^34^

So far we have seen that Cheek's interests, as expressed in his contributions to the Plinian Society, were eclectic to say the least, ranging from hydrology to invertebrate zoology and antiquities. There are, however, two pieces of evidence from the minutes that suggest a possible interest in transformism. The first relates to his participation in a discussion that followed Browne's ‘remarks on the question whether any change might be produced on vegetables sufficient to constitute new genera and species’ in a paper on the botany of the Edinburgh area read on 18 December 1827.^35^ In the absence of any record of what exactly was said in this discussion, this in itself gives no clue as to Cheek's attitude to transformism, although it does indicate an interest in the subject. The evidence of a communication from Cheek to the society on 5 April 1831 is a little more compelling. This was his last such communication, although his last recorded attendance was in fact not until 3 April 1832. In this communication he recounted a case in which a ‘pointer bitch had had her tail mutilated in a trap or by some such accident, in a litter of three pups she produced shortly after they all had tails with a less number of caudal vertebrae than the species in general have.’^36^ This is a clear example of the inheritance of acquired characteristics, implicated by both Lamarck and Erasmus Darwin (1731–1802) as an important mechanism in the formation of new species. Of course, Cheek may simply have seen the case of the dog's tail as a curiosity of natural history, so too much weight should not be given to it as evidence of Cheek's views on transformism. However, much more compelling evidence emerges from the records of the Royal Medical Society and from the pages of the *Edinburgh Journal of Natural and Geographical Science*.

## Cheek at the Royal Medical Society of Edinburgh

The Royal Medical Society was founded in 1737 for medical students at the University of Edinburgh and is still in existence today. Cheek joined the society on 3 November 1826.^37^ (Darwin joined on 17 November of the same year.) The society holds a complete set of minute books going back to its foundation. These, however, record only the private business of the society, dealing principally with issues of membership, finance and dealings with other societies. They do not provide details of debates that took place or the attendance of individual members. The society's library also holds a complete set of dissertations read to the society, which in contrast is an extremely valuable source of information on the interests of the society's members.

A widely debated topic of the period, the ‘varieties of man’, was one of the more popular non-medical subjects for dissertations. Six dissertations were presented to the Royal Medical Society on the subject between 1800 and 1836. Of these, five supported the monogenist view that all the races had a common origin, against only one polygenist essay. In his contribution to the debate, read before the society on 29 January 1830, Cheek supported the more popular monogenist view but went well beyond simply proposing a common origin for the varieties of the human race. He questioned the distinction between species and varieties, and the very existence in nature of species as conventionally understood. He went as far as to propose a common origin for all species. First he tackled the species concept itself, admitting that

I have not met with an author who can distinguish the species from the permanent variety. And Buffon probably was not so widely inaccurate, when he said ‘qu'il n y a pas d'espece dans la Nature’, for if an animal be liable to the casual production of varieties, whose new characters are transferable to its offspring, these definitions of species must either be declared to be inexact, or the nonexistence of species must be admitted.^38^

As well as reflecting views expressed by the Comte de Buffon (1707–88), this also accords with the view on the unreality of species adopted by Lamarck, who believed that species had no real existence in nature, as living things gradually transformed into entirely new forms over geological time.^39^ All classification was therefore an artificial creation of natural historians, which was useful only from the perspective of one particular moment in the history of the Earth.

Cheek then addressed the question of the distinction between species and varieties. First he noted that the conventional distinction was based on ‘the *origin* of the difference’. If we imagine that we can find a natural cause for the difference between two types of living thing—presumably Cheek is thinking principally of the effect of the environment here—we name them varieties; if not, we must assume that the difference has existed *ab initio*, and we name them species. As Cheek himself put it: ‘If we fancy we can devise a probable cause for a particular diversity, we name it a variety. If mystery overpowers our subtlety, we name it species.’^40^ Cheek therefore denied that there was an intrinsic difference between species and varieties, and went further to suggest, as had Buffon and Lamarck before him, that species had no real existence in nature. His refusal to recognize that there were limits to the mutability of species opened the door for the possibility of the transmutation of species.

Having cast doubt on the distinction between species and varieties, Cheek then turned to the question of how both arose. He summarized the various possibilities as he saw them in a remarkable passage that merits quotation at length:

All the varieties, as well as all species, may have been transmitted from a single pair, by spontaneous changes; or some physical alterations in the constitution or revolutions of the globe, and its atmosphere may have produced them from a specific type, as some similar changes may have caused the differences of species, whilst previously their several characters were associated under a genus; or lastly the characteristics of permanent varieties, or rather of species may have appeared at once over all those portions of the globe, were the necessary conditions of existence assembled. But the subject in the present state of our knowledge, is a mere barren waste of speculation, where all labour is misspent, & the seed falls upon a rock. I am myself inclined to the opinion that the accepted laws of cause & effect are not applicable to the discussion of questions on organization—that the climate does not *cause* the change—but that there is an ultimate Zoological law, that structures have a tendency to change for adaption to new functions—that the indigenous races are adapted to the climates in which they are found—and that, if a race be removed to a new region it will either become adapted to the new functions required, by the powers or organization, or that it will propagate a sickly & imbecile offspring, and ultimately perish.^41^

Here Cheek gives us three possible theories for the origin of species.^42^ First, he considers the possibility that ‘spontaneous changes’ may be responsible for the diversity of life. This implies that living things have an innate tendency to change their forms over time. Second, he suggests that species may have been moulded directly by changes in their environments, so as to generate new species as their conditions of life change. Third, Cheek considers the possibility that species and varieties came into being spontaneously wherever the environment was suitable for them. The first model is close to Lamarckian transformism, in which the principal mechanism for the transmutation of species was an innate tendency to increasing perfection, although Lamarck did recognize that changes resulting from environmental pressures could complicate this simple progressive pattern.^43^ The second is closer to the theories of Étienne Geoffroy Saint-Hilaire (1772–1844), who proposed that directional change in the environment, in this case in the composition of the atmosphere, led to progressive changes in living things. The third theory is essentially the model proposed by Buffon in his *Époques de la Nature* (1778), where he suggested that as the Earth cooled over time, new species arose that were adapted to the cooler conditions, while warmth-loving species became extinct. Although Buffon gave no indication of how he thought this came about, he clearly was not proposing the transmutation of species but rather the appearance *de novo* of entirely new species. Curiously, Cheek then appeared to dismiss the whole question as a ‘mere barren waste of speculation’ before giving his own opinion. Was this an attempt to deflect criticism from his own opinions by denying the validity of any such theorizing? It is impossible to say for certain.

When he did give his own view, he presented a model that was much closer to that of Geoffroy. Like Geoffroy, he suggested that the environment, although not causing adaptive change directly, did exert a selective pressure, eliminating those forms that were ill-adapted to the environment in which they found themselves. The transmutations themselves, on which the selective action of the environment could operate, occurred through the action of a ‘Zoological law’, which was manifested as an innate tendency to produce new variations in living things. This is very close to Geoffroy's theory:

The insensible changes [in the atmosphere] from one century to the next finish by reaching a certain value, at which point respiration becomes difficult and finally impossible for certain organ systems: it therefore requires and creates for itself another arrangement, perfecting or altering the pulmonary cells in which it operates; modifications which may be beneficial or harmful. These then propagate and spread through the rest of the animal economy. If those modifications lead to harmful effects, the animals which undergo them will cease to exist, to be replaced by others, with forms modified to suit the new circumstances.^44^

The paragraph quoted from Cheek's essay above is followed by this enigmatic passage:

The question as to the unity of origin is not necessarily connected with the inquiry as to identity of species. Founded on historical & traditional considerations, the supposition of a single origin for every species does not partake of the nature of a fact to be admitted in Zoology. I believe it to be impossible, and am prepared with my proofs.^45^

Taking ‘single’ to mean ‘separate’, this would seem to be a strong affirmation of Cheek's belief that all species have a common origin, and that the contrary opinion was only the result of erroneous ‘historical and traditional’ beliefs. However, taking ‘every species’ to mean ‘all species’, he would seem to be saying the opposite. Taken in the context of what has gone before, the first interpretation seems the more likely, although Cheek seems to have chosen to express himself in a way that is curiously opaque and ambiguous. Of the proofs he had prepared, he sadly had no more to say. Cheek then ended the dissertation on a curiously disconsolate note, remarking, ‘I am so much dissatisfied at the mode in which I am obliged to treat this problem which I have selected from a transcendental philosophy that it will be better now to terminate.’^46^ Who or what had obliged him to treat the problem in a way that he seemed to find so unsatisfying is unfortunately left to the imagination of the reader. Cheek's dissertation raises as many questions as it answers. What we are not left in doubt about is that Cheek was a convinced transformist.

To sum up the evidence of Cheek's dissertation for the Royal Medical Society, this intriguing document suggests that his ideas were influenced by the theories of Lamarck and Geoffroy, as well as by the older writings of Buffon, when formulating his own opinions on the transmutation of species. In his dissertation he briefly outlined the three major theories of the history of life that he had considered: first, that living things had an innate capacity for progressive change from generation to generation; second, that changes in the environment caused the transmutation of species; and third, that entirely new species arose spontaneously as a consequence of environmental change. These three possibilities represent highly condensed versions of the respective theories of Lamarck, Geoffroy and Buffon. In the end, Cheek settled on a model close to that of Geoffroy.

## The *Edinburgh Journal of Natural and Geographical Science*

While a student at Edinburgh, Cheek edited a short-lived journal entitled the *Edinburgh Journal of Natural and Geographical Science*. For the first two volumes his co-editor was William Francis Ainsworth, his friend, fellow medical student and fellow member of the Plinian Society. The final volume was edited by Cheek alone, with editorial assistance in specific areas provided by Sir William Jardine, G. A. Walker Arnott, John Scouler, Robert Knox and J. F. W. Johnston, who had ‘undertaken the entire direction of their several Departments’.^47^ In total, 21 monthly issues of the journal were produced. It was published in Edinburgh and also distributed in London and Dublin. According to its title page, the third volume was in addition distributed in Paris.^48^ Reviews appeared in at least six periodicals, five of them based in Edinburgh, although none of the major quarterly reviews devoted any attention to it.^49^ This journal published articles on a wide variety of scientific and geographical subjects, including some by such notable Edinburgh naturalists as William Macgillivray, Sir William Jardine and John Fleming. Despite the youth and inexperience of its founders, the journal therefore seems to have had the support of several important figures in Edinburgh natural history. It also provided Cheek with a platform for his transformist views.

Cheek used his journal to give a decidedly partisan commentary on the famous and acrimonious debate of 1830 at the Academy of Science in Paris on unity of plan that took place between Geoffroy, the great champion of the concept, and Georges Cuvier, its most famous opponent.^50^ Geoffroy had come to believe that the anatomies of all animals were variations on one ideal body plan, a theory that Cuvier totally rejected. The immediate cause of the debate was Geoffroy's support for the claim that the unity of plan he had established among vertebrates could be extended to molluscs. This was too much for Cuvier, who set out to refute Geoffroy's theories once and for all. Although Geoffroy was a transformist, and Cuvier a strong opponent of the transmutation of species, the debate was conducted strictly on the terrain of unity of plan, without straying into related areas of contention. The confrontation between the two great comparative anatomists became something of a *cause célèbre* in intellectual circles across Europe and was widely reported. The *Edinburgh Journal of Natural and Geographical Science* devoted considerable space to ‘a controversy which has arisen between the two first zoologists of the age’.^51^ The April number contained a detailed account of Cuvier's arguments against Geoffroy. In May followed Geoffroy's answer to Cuvier's charges. According to Toby A. Appel, ‘even the supporters of Geoffroy agreed that Cuvier had had the upper hand in the debate’ and that ‘Cuvier could be said to have won the day’.^52^ Although the articles in Cheek's journal evince a healthy respect for both of the great comparative anatomists, the reader is left in little doubt that the author favours Geoffroy's arguments. Rather than acknowledge Cuvier the victor, the second article reports that ‘M. St. Hilaire has consented to relinquish the discussion in the Academy; but, confident in the truth and novelty of his conclusions, he has determined to write a work, wherein he will controvert the opinions of M. Cuvier.’^53^ In the event, Geoffroy's *Principles of Philosophical Zoology*, rushed into print in April 1830, did not definitively put an end to the dispute. Only the death of Cuvier in May 1832 finally prevented any further continuation of hostilities. There is little doubt where Cheek's sympathies lay, and his espousal of Geoffroy's principle of unity of plan, as well as his transformist theories, are evident elsewhere in Cheek's journal.

In an article on the dugong in the December 1829 issue of the journal, Cheek seemed to combine a developmental vision of the history of life with the concept of unity of form associated with the philosophical anatomy of Geoffroy. In this article he noted that dugongs are ‘nothing else than terrestrial mammalia, whose internal organs are concealed under the figure of a fish.’ He went on:

Speculation immediately suggests the geological fact, that fishes existed prior to the creation of the mammalia; and that the Omnipotent has passed by slow gradations from one series of organization to another; that the type or model on which all vertebrate animals are formed is essentially the same.^54^

That nature had brought forth species progressively over time in an ascending scale of perfection does not necessarily imply the transmutation of species. However, the fact that Cheek commented on the slow rate at which the series of organization pass into each other strongly implies that it was a process of transmutation that he had in mind. It would have made little sense to talk about the rate at which each series had passed into the next if each had been an entirely separate creation.

Another reference to unity of type is found in the March 1831 issue in an anonymous editorial that postulates the identity of the vascular arches of terrestrial vertebrates with the branchial arches of fish. This article, almost certainly by Cheek, is contained in a section of the journal entitled ‘Zoological Collections’. Here it is noted that

the transcendental anatomists infer, that the vertebrate (if not the invertebrated) animals are constructed according to the same *type* or plan; and that the higher animals, before arriving at their ultimate degree of development, successively run through stages in which their structure is similar to that of animals of less complex organization.^55^

It is, however, quickly pointed out that this does not mean that the fetus of a human being actually is a fish, a reptile or a bird at any stage in its development, because not all the organs pass through the same stage of development at the same time. The article goes on to refer to Geoffroy's 1829 transformist article from the *Mémoires du Muséum d'Histoire Naturelle*, noting that from ‘this gradual process of formation we can understand the production of monsters, some of which have been shown by St Hilaire to be caused by stoppage of the development of some of their parts’.^56^ Given the evidence of his articles in the *Edinburgh Journal of Natural and Geographical Science*, Cheek, like his older contemporaries in Edinburgh medical circles, Grant and Knox, was almost certainly a disciple of Geoffroy, subscribing to both his theories of unity of plan and of transformism. It may well have been through either Knox or Grant, a fellow member of the Plinian Society, that Cheek was first exposed to these ideas.

In the April 1830 issue of the journal an article entitled ‘Suggestions on the relation between Organized Bodies, and the Conditions of their Existence’ appeared in the section of the journal called ‘Natural-Historical Collections’, which was composed of miscellaneous short articles on subjects of natural-historical interest, most of them unattributed. The article was anonymous but was again almost certainly by Cheek, given its similarity in tone and language to his Royal Medical Society essay and other articles that he published under his own name. (His co-editor Ainsworth is unlikely to have been the author because, unlike Cheek, he is not known to have taken a significant interest in such matters.) The ‘conditions of existence’ were first defined as ‘the external physical agents with which the organized body is in necessary relation, and upon which the integrity and action of its functions depends’.^57^ This is a term often associated with Cuvier, for whom ‘conditions of existence’ were synonymous with ‘final causes’.^58^ The term was also used by Geoffroy, but for him the conditions of existence became an efficient cause.^59^ It is most probably from the latter source that Cheek borrowed it. Cheek's article went on to explain the relationship between living things and their conditions of existence in the following seven propositions, which merit quotation in full:

(1) The development of the process of organization,—a power imposed by the Deity upon matter,—depends upon the conditions of existence.

(2) The perfection of organized bodies, or the number and complexity of organs, has a direct ratio with the number of the conditions of existence.

(3) All organized bodies possess the power of varying the development of the organs, by addition or subtraction of parts, as changes in the conditions of existence occur.

It is easy to conceive that an organized body can assimilate elements in the form of a new organ, as new functions are required, when we recollect that it is constantly exercising a power of converting inorganic matter into the living emblem of its original form.

(4) The characters of organized bodies will be permanent during the continuation of the same conditions of existence which led to their development, and no longer.

(5) The more numerous the conditions of existence, the less liable the characters of the organized body to change, and *vice versa*.

(6) It has been observed that the older formations of the earth's crust, generally speaking, the less perfect the organic remains they contain. This progressive increase of perfection of organization, would lead us to expect, from the foregoing principle, that, with the advancing age of the earth, the conditions have increased in number; and this seems to be a fact.

(7) Adaptation of the law by which organized bodies change with the variation of the conditions of existence; and separation of the functions of relation, and concentration of the vital functions, seems to be the mode of perfection.^60^

Although some of this seems rather obscure, it would appear undeniably to be an unambiguous theoretical statement of the principle that the development of the organization of living beings is directed by their environment, in a manner broadly in harmony with the theories of Geoffroy. Cheek even went on to link this to the increasing perfection of living forms in the course of geological time. It is worth noting that the idea expressed in his fourth proposition, which states that living things will remain the same as long as their external environment remains unchanged, is identical to a concept developed in Geoffroy's transformist article on gavials, published in 1825, and may be derived directly or indirectly from that source.^61^ On the basis of the evidence of the second and fifth propositions, Cheek's theory also seems to have implied that the more that conditions of existence acted on living things, the more perfect they became but the less was further change possible. It is not entirely clear how Cheek envisaged this process, but it seems likely that he imagined that over geological time the web of relations between an organism and its physical and biological environment would become more complex. As more conditions came into play over time, the progress of living things towards greater perfection would slow down, and Cheek saw evidence for this in the fossil record.

It is furthermore suggested in proposition three that new organs appear, or are lost, in response to changes in the environment. This would clearly seem to be a concept related to the principle of the development or disappearance of organs through use or disuse that is central to Lamarck's transformist thought. Lamarck wrote in his *Philosophie Zoologique* that ‘the failure to use an organ, become constant because of the habits which have been adopted, gradually reduces the organs, and finishes by making it disappear.’^62^ Conversely, ‘the frequent use of an organ which has become constant through habit, augments the faculties of that organ.’^63^ These changes are then transmitted to offspring. Here, as elsewhere, we see Cheek drawing on the work of contemporary transformist thinkers to produce his own synthesis.

The subject of the inheritance of acquired characteristics, raised by Cheek at the Plinian Society in April 1831, was raised by him again in two articles published in March and May 1831 in the *Edinburgh Journal of Natural and Geographical Science*, in which the transmission of mutilations suffered by animals to the next generation was discussed in the context of the theories of Johann Friedrich Meckel (1781–1833) and Johann Friedrich Blumenbach (1752–1840).^64^ Cheek was, however, sceptical that such a crude modification of an animal as the loss of a tail could be transmitted from generation to generation in the absence of a more lasting change in its conditions of existence:

We have, in a former number, stated it to be our opinion, that the characters of an organism are permanent during the operation of the circumstances, internal or external, which produce them, *and no longer*. Whence, the original deficiency of caudal vertebrae being the result of mutilation, a new operation would be required in every successive generation, to continue the character (if it may be so called) in the race.^65^

It can be inferred from the above that Cheek saw the inheritance of acquired characteristics as unnecessary to explain the transmutation of species, and that the increasing complexity of living things over time was both driven and maintained directly by the increasing complexity of the conditions of existence. Here again he deviated from the Lamarckian model of transformism, and was closer to Geoffroy.

## Conclusions

A small but significant number of members of the Plinian Society read papers or communications on topics related to the transmutation of species, spontaneous generation and the materialistic theories of life and mind; these included William A. F. Browne, William Rathbone Greg and Thomas Shapter. But in most cases we know nothing more of their thoughts on the subject. Even the evidence we have from the minutes of the Plinian Society is often too brief to give a clear picture. In contrast, we have seen that Cheek's tempestuous but highly productive career at the University of Edinburgh has left us with a significant body of evidence regarding his opinions on one of the great topics of the day, the transmutation of species, going well beyond the sketchy evidence of the Plinian Society's minute books.

What this evidence seems to show is that for Cheek, as for Geoffroy, it was change in the conditions of existence that was the driving force behind evolutionary change. Although he was interested in the phenomenon of the inheritance of acquired characteristics, he seems to have discounted it as an important mechanism in the transmutation of species. For Cheek, variation occurred as a result of the operation of a ‘Zoological law’, which generated variations. However, these changes would go nowhere without the selective pressure to adapt to changing conditions of existence. Under the influence of such changes, new organs could come into being, and old ones that are no longer required could be lost. As in Geoffroy's theory, these changes in conditions of existence were the result of physical changes on the Earth's surface, relating to a variety of factors including the composition of the atmosphere.

We have seen that in the years during which Darwin was an undergraduate at Edinburgh it was possible for a medical student to assemble quite a sophisticated and original theory of the transmutation of species based on the ideas of a variety of recent and contemporary European transformist thinkers that were evidently circulating in Edinburgh at this time. Darwin later implied that he had not been influenced by the transformist ideas he encountered in Edinburgh. Anxious, perhaps, to be seen to have adhered to the inductive method that dominated scientific discourse in the mid nineteenth century, he showed little enthusiasm for crediting his transformist predecessors, who were widely condemned for their fanciful speculations. This would have been particularly true in the aftermath of the fiercely hostile reception given to Robert Chambers's *Vestiges of the Natural History of Creation* (1844) in many scientific circles.^66^ It was not until the third edition of *Origin of Species* in 1861 that he added ‘An historical sketch of the recent progress of opinion on *The Origin of Species*’, listing some earlier evolutionary thinkers. Grant, who was still alive and lecturing in comparative anatomy at University College London at the time, could not be completely ignored but merited only a scant paragraph that did not mention that Darwin had ever known him personally.^67^ In his ‘Recollections’, written in 1876, he denied that his friendship with Grant, an active advocate of transformism in those years, had had any influence on his ideas, claiming that when Grant ‘burst forth in high admiration of Lamarck’, he ‘listened in silent astonishment, and as far as I can judge, without any effect on my mind.’^68^

There is in fact significant evidence that Grant and Darwin became close friends after the latter's brother Erasmus left Edinburgh in 1826, and that they spent much time together on collecting trips along the shores of the Firth of Forth.^69^ We know that Grant had made reference to the *Zoonomia* of Darwin's grandfather Erasmus in an undergraduate dissertation in 1814.^70^ Much later, in the dedication to Darwin in Grant's *Tabular View of the Primary Divisions of the Animal Kingdom* (1861), he wrote, ‘More than fifty year have now elapsed since the “Zoonomia” of your illustrious ancestor, Dr. Erasmus Darwin, first opened my mind to some of “the laws of organic life”.’^71^ It would be astonishing if Grant had never mentioned the influence of Erasmus Darwin on him to his grandson during their collecting trips, but Darwin made no mention of this, or of any talk of Lamarck other than on the one solitary occasion noted above. It is perhaps no surprise that he also made no mention in his autobiographical writings of other transformists whom he may have become acquainted with in Edinburgh. Yet Darwin had been surrounded by the same ferment of ideas as Cheek in 1826–27, mixed socially with the same group of medical students, attended the same lectures and was a member of the same societies.^72^ It is hard not to suspect that Darwin was being somewhat disingenuous when he claimed that his theory of evolution owed nothing at all to his time in Edinburgh.

## Notes

1 Adrian Desmond and James Moore, *Darwin*, p. 31 (Penguin, London, 2009).

2 *Ibid*., p. 41.

3 Janet Browne, *Charles Darwin: voyaging*, p. 77 (Pimlico, London, 2003).

4 I will generally use the terms ‘transmutation of species’ or ‘transformism’ in this paper to distinguish these older theories from Darwinian evolution, from which they vary significantly. The term ‘evolution’ was current in the 1820s but was generally used with reference to fetal development and was often associated with preformationist theories of generation.

5 See, for example, Adrian Desmond, *The politics of evolution: morphology, medicine and reform in radical London* (Chicago University Press, 1989); James A. Secord, ‘Edinburgh Lamarckians: Robert Jameson and Robert E. Grant’, *J. Hist. Biol*. **24**, 1–18 (1991); Evelleen Richards, ‘The “moral anatomy” of Robert Knox: the interplay between biological and social thought in Victorian scientific naturalism’, *J. Hist. Biol*. **22**, 373–436 (1989).

6 Secord, *op. cit*. (note 5).

7 Online Parish Clerks for the County of Lancashire, Baptisms at St Stephen in the City of Salford, Baptisms recorded in the register for the years 1794–1812 (source: Latter Day Saints (LDS) file 1408727) (see http://www.lan-opc.org.uk/indexp.html). Unfortunately the exact dates of birth and death cannot be established, because before the beginning of civil registration in 1837 we are dependent on parish records, which give only the dates of baptism and burial for Cheek.

8 William Henry Cheek, solicitor, of Tideswell, Derbyshire (Derbyshire Record Office ref. no. D5480) (see http://calmview.derbyshire.gov.uk/calmview/Record.aspx?src=CalmView.Catalog&id=D5480&pos=1 (accessed 4 October 2014)).

9 Henry H. Cheek, *An Answer to Certain Statements Contained in Mr Neill's ‘Address to the Members of the Wernerian Natural History Society’. 25 September 1830*, pp. 3–4 (Edinburgh, 1830).

10 *Ibid*., p. 4.

11 Patrick Neill, *Supplement to an Address to the Wernerian Natural History Society, Dated July 1830; Containing a Reply by Mr Neill to Mr Cheek's Answer. November 1830*, pp. 15–16 (Edinburgh, 1830).

12 For an excellent account of this controversy, see Forbes W. Robertson, *Patrick Neil: doyen of Scottish horticulture*, pp. 46–47 (Whittles Publishing, Dunbeath, 2011).

13 Online Parish Clerks for the County of Lancashire, Burials at St Stephen in the City of Salford, Burials recorded in the register for 1813–1838, Burials for 1831–1838 (source: Manchester Central Library) (see http://www.lan-opc.org.uk/indexp.html).

14 William Baird, *Memoir of the Late Rev, John Baird, Minister of Yetholm, Roxburghshire; with an Account of his Labours in Reforming the Gypsy Population of that Parish*, p. 63 (London, 1862).

15 Anon, ‘Natural History in Scotland’, *Mag. Nat. Hist. J. Zool. Bot. Mineral. Geol. Meteorol.* **1**, 291–292 (1829).

16 Browne, *op. cit*. (note 3), p. 73.

17 Scottish Universities Commission (1826), *Minutes of evidence taken before the Commissioners for visiting the universities and colleges in Scotland: University of Edinburgh 1826, 1827, 1830*, p. 92 (Edinburgh?, 1830?).

18 *Ibid*.

19 Minutes of the Plinian Society, 1826–1841, vol. 1 (Library of the University of Edinburgh Dc.2.53), f. 25 verso.

20 See, for example, Howard E. Gruber, *Darwin on man: a psychological study of scientific creativity*, p. 39 (Wildwood House, London, 1974); Desmond and Moore, *op. cit*. (note 1), p. 38; Browne, *op. cit*. (note 3), p. 77.

21 Minutes of the Plinian Society, 1826–1841, vol. 2 (Library of the University of Edinburgh Dc.2.54), f. 5 verso.

22 Anon, *op. cit.* (note 15).

23 Plinian Society, *op. cit*. (note 21), f. 98 recto.

24 *Ibid*., f. 99 verso.

25 *Ibid*., f. 125 verso.

26 Plinian Society, *op. cit*. (note 21), f. 35 recto.

27 *Ibid*., f. 46 recto.

28 *Ibid*., f. 55 recto.

29 Charles Newland, ‘Observations on the milky appearance of spots of water in the sea’, *Phil. Trans.* **62**, 93–94 (1772). (doi:10.1098/rstl.1772.0014)

30 Plinian Society, *op. cit*. (note 19), f. 59 verso.

31 *Ibid*., f. 87 verso.

32 *Ibid*., f. 105 verso.

33 *Ibid*., f. 114 recto.

34 *Ibid*., f. 115 verso.

35 *Ibid*., f. 85 verso.

36 Plinian Society, *op. cit*. (note 21), ff. 90 verso–91 recto.

37 The Royal Medical Society of Edinburgh, *List of Members*, p. 44 (Royal Medical Society of Edinburgh, Edinburgh, 1906).

38 Henry Cheek, ‘On the varieties of the human race’, Royal Medical Society, Dissertations 91 (1829–30), p. 301 (Library of the Royal Medical Society).

39 Jean-Baptiste Lamarck, *Philosophie Zoologique*, vol. 1, p. 74 (Dentu, Paris, 1809).

40 Cheek, *op. cit*. (note 38), p. 302.

41 *Ibid*., pp. 306–307.

42 Although Cheek doubted the existence of species in nature he continued to use the term in his writings, presumably for want of a suitable alternative. I have followed his example in this paper when discussing his theories.

43 Lamarck, *op. cit*. (note 39), pp. 206–207.

44 ‘Les modifications insensibles d'un siècle à un autre finissent par s'ajouter et se réunissent en une somme quelconque ; d'où il arrive que la respiration devient d'une exécution difficile et finalement impossible quant à de certaines systèmes d'organes : elle nécessite alors et se crée è elle-même un autre arrangement, perfectionnant ou altérant les cellules pulmonaires, dans lesquelles elle opère ; modifications heureuses ou funestes, qui se propagent et qui influent dans tout le reste de l’économie animal. Car si ces modifications amènent des effets nuisibles, les animaux que les éprouvent cessent d'exister, pour être remplacés par d'autres, avec des formes un peu changées, et changées à la convenance des nouvelles circonstances.' Étienne Geoffroy Saint-Hilaire, ‘Quatrième mémoire, lu à l'académie des sciences, le 28 mars 1831’, *Recherches sur de Grands Sauriens Trouvés à l'État Fossile vers les Confins Maritimes de la Basse Normandie, Attribués d'abord au Crocodile, puis Déterminés sous les Noms de Téléosaurus et Sténosaurus*, p. 79 (Paris, 1831).

45 Cheek, *op. cit*. (note 38), p. 307.

46 *Ibid*.

47 Title page, *Edinb. J. Nat. Geogrl Sci.* **3**, 1 (1831).

48 The title pages of the first and second volumes list as publishers and distributors Daniel Lizars in Edinburgh, Whittaker, Teacher, & Arnot in London and William Curry & Co. in Dublin. For the third and final volume John Anderson replaced Lizars in Edinburgh, and J. B. Baillière in Paris had been added.

49 The editors quoted reviews from the following periodicals in the front matter to volume 1: *Edinburgh Literary Gazette* (October 1829); *Edinburgh Observer* (October 1829 and February 1830); *Edinburgh Evening Post* (March 1830); *North Briton* (March 1830); and *Edinburgh Literary Journal, or, Weekly Register of Criticism and Belles Lettres* (January 1830 and April 1831). Positive reviews also appeared in *Monthly Magazine, or, British Register* (July 1829) and *Mirror of Literature, Amusement and Instruction* (October 1829).

50 For a comprehensive and detailed account of the debate and its impact, see Toby A. Appel, *The Cuvier–Geoffroy debate: French Biology in the decades before Darwin* (Oxford University Press, New York, 1987).

51 [Henry Cheek], ‘Review of the recent discussion before the Academy of Sciences in Paris, on the “unity of organization”. Part I,—Baron Cuvier's Views’, *Edinb. J. Nat. Geogrl Sci.* **2**, 37 (April 1830).

52 Appel, *op. cit*. (note 50), p. 171.

53 [Henry Cheek], ‘Review of the recent discussion before the Academy of Sciences in Paris, on the “unity of organization”. Part II,—M. Geoffroy St. Hilaire's answer to Baron Cuvier’, *Edinb. J. Nat. Geogrl Sci.* **2**, 116 (May 1830).

54 Henry Cheek, ‘On the Natural History of the Dugong, (*Halicore Indicus*, Desm.)—the Mermaid of Early Writers, and particularly on the differences which occur in its Dental Characters’, *Edinb. J. Nat. Geogrl Sci.* **1**, 162 (December 1829).

55 [Henry Cheek], ‘On the Existence of Vascular Arches in the Foetus of Mammifera, Birds, and Reptiles, similar to the Branchial Arteries in Fishes and the Larvae of the Batrachian Reptiles’, *Edinb. J. Nat. Geogrl Sci.* **3**, 235 (April 1831).

56 *Ibid*., p. 235.

57 [Henry Cheek], ‘Suggestions on the relation between Organized Bodies, and the Conditions of their Existence’, *Edinb. J. Nat. Geogrl Sci.* **2**, 65 (1830).

58 See, for example, Georges Cuvier, *Le Règne Animal Distribué d'après son Organisation*, vol. 1, p. 6 (Deterville, Paris, 1817).

59 See, for example, Étienne Geoffroy Saint-Hilaire, ‘Anencéphales humains’, *Mém. Mus. Hist. Nat.* **12**, 267 (1825).

60 [Cheek], *op. cit*. (note 57).

61 Etienne Geoffroy Saint-Hilaire, ‘Recherches sur l'organisation des gavials’, *Mém. Mus. Hist. Nat.* **12**, 97–155 (1825).

62 ‘Le défaut d'emploi d'un organe, devenu constant par les habitudes qu'on a prises, appauvrit graduellement cet organe, et fini par le faire disparaître’. Lamarck, *op. cit*. (note 39), p. 220.

63 ‘L'emploi fréquent d'un organe devenu constant par les habitudes, augmente les facultés de cet organe’. *Ibid*., p. 225.

64 Anon, ‘Query on the Hereditary Transmission of Accidental Characters’, *Edinb. J. Nat. Geogrl Sci.* **3**, 173 (1831); B. S. Shuttleworth, ‘Hereditary Transmission of Accidental Characters’, *Edinb. J. Nat. Geogrl Sci.* **3**, 301 (1831).

65 Henry Cheek, editorial comment on ‘Query on the Hereditary Transmission of Accidental Characters’, *Edinb. J. Nat. Geogrl Sci.* **3**, 173 (1831).

66 See, for example, Secord, *op. cit*. (note 5).

67 Charles Darwin, *The Origin of Species by Means of Natural Selection, or the Preservation of Favoured Races in the Struggle for Life*, 3rd edn, p. xiv (John Murray, London, 1861).

68 Charles Darwin, ‘1876 May 31—Recollections of the development of my mind and character’, in *Autobiographies*, p. 24 (Penguin, London, 2002).

69 Browne, *op. cit*. (note 3), pp. 80–82.

70 Robertus E. Grant, *Dissertatio Physiologica Inauguralis, de Circuitu Sanguinis in Foetu* (Edinburgh, 1814), p. 8.

71 Robert Edmond Grant, *Tabular View of the Primary Divisions of the Animal Kingdom, Intended to Serve as an Outline of an Elementary Course of Recent Zoology*, p. v (Walton & Maberly, London, 1861).

72 For a broader overview of the Franco-Scottish influence on Darwin's thought, see M. J. S. Hodge, ‘On Darwin's science and its contexts’, *Endeavour*, http://dx.doi.org/10.1016/j.endeavour.2014.10.003 (published online 6 November 2014). Another important recent contribution to our knowledge of the significance of Scottish channels for the dissemination of French science in Britain in this period is Jonathan R. Topham's ‘Science, print and crossing borders: importing French science books into Britain, 1789–1815’, in *Geographies of nineteenth-century science* (ed. David N. Livingstone and Charles W. J. Withers), pp. 311–344 (University of Chicago Press, 2011).

